# The Effectiveness of self management program on quality of life in patients with sickle cell disease 

**Published:** 2015-03-15

**Authors:** M Ahmadi, S Jahani, S Poormansouri, A Shariati, H Tabesh

**Affiliations:** 1 Lecturer in Nursing and Midwifery, Department of Nursing, Nursing and Midwifery School, Ahvaz Jundishapur University of Medical Sciences, Ahvaz, I.R. Iran.; 2 Lecturer in Nursing and Midwifery, Department of Nursing, Nursing and Midwifery School, Ahvaz Jundishapur University of Medical Sciences, Ahvaz, I.R. Iran.; 3 MS.c student in Nursing, Department of Nursing, Nursing and Midwifery School, Ahvaz Jundishapur University of Medical Sciences, Ahvaz, I.R. Iran.; 4 Lecturer in Nursing and Midwifery, Department of Nursing, Nursing and Midwifery School, Ahvaz Jundishapur University of Medical Sciences, Ahvaz, I.R. Iran.; 5 Assistant Professor in Biostatistics and Epidemiology, Department of Biostatistics and Epidemiology, Faculty of Health, Ahvaz Jundishapur University of Medical Sciences, Ahvaz, I.R. Iran.

**Keywords:** Self-management, Quality of life, sickle cell

## Abstract

**Background:**

Sickle cell patients suffer from many physical, psychological, and social problems that can affect their quality of life. To deal with this chronic condition and manage their disease and prevent complications associated with the disease, they must learn skills and behaviours. The aim of this study was to determine the effectiveness of self-management programs on quality of life in these patients.

**Material and Methods:**

Samples of this quasi-experimental study, which included 69 patients with sickle cell disease referring to the Thalassemia Clinic of Shafa Hospital, were entered into the study by census method. Patients received a self-management program using the 5A model for 12 weeks, while their quality of life before the intervention were assessed at the twelfth week and thirty-sixth week using SF-36 questionnaire. Data were analyzed by descriptive statistics, paired t-test, Wilcoxon test, Hotelling's T2, and repeated measures test.

**Results:**

The eight dimensions and the total QoL score after intervention were significantly increased compared to those before the intervention (P<0.001). Repeated measures test showed that the mean score of eight QoL dimensions and the total QoL score decreased in the thirty-sixth week, compared to twelfth week. However, it was significantly enhanced in comparison with the intervention baseline (P<0.05).

**Conclusions:**

Current study revealed the efficacy of self-management interventions on the quality of life in patients with sickle cell disease. Therefore, application of this supportive method could be useful to empower the patients and help them to manage the disease.

## Introduction

Sickle cell disease is a genetic disorder of haemoglobin that affect millions worldwide (1). 300,000 infants are born annually with this disease and there are approximately 2.5 million people in the United States and 300 million in the world who have sickle cell trait today (2,3). According to the latest statistics, this disease which is found in the southern provinces of Iran, especially Khuzestan, has affected approximately 500 sickle cell patients in the Khuzestan province (4). Complications of the disease can be severe and life-threatening, including anemia, stroke, pulmonary dysfunction, major organ complications, and unexpected and chronic pain crises. These are the most common symptoms reported by the patients, causing repeated referrals to the emergency department or hospital to receive medical care in the patients (5-7). 

Although sickle cell patients experience high mortality at young ages, the use of prophylactic antibiotics, such as penicillin, vaccines, and treatments reduce the severity of disease such as hydroxyurea have increased the average life span of these patients up to 50 years (6,8). Despite the increase in life expectancy, decline in some areas of health can be observed in patients. Unexpected and chronic pains, repeated referrals to the emergency department and hospital as well as unemployment have led sickle cell patients to lower self-esteem, feelings of frustration (9), depression (9-11), anxiety and stress (10), and poor quality of life (12-15).

Self-management seems to be essential to improve quality of life and health status of sickle cell patients. To achieve an acceptable level of quality of life, they need to learn how to manage and control the disease (16). When a sickle cell disease is diagnosed, the affected people need a comprehensive and organized care, including medical and non-medical services as well as self-management strategies (17). 

Since learning strategies to cope with the disease, which only comply with the pharmaceutical principles, cannot lead to increased skill in dealing with the disease at home and in the community (18), a shift from a palliative medical model to a participatory prevention-based approach may be involved in reducing unnecessary medical costs for these patients (19). The acquisition or modification of effective coping strategies, can lead to a reduction in symptoms, promote self-management behaviors and health outcomes and achieve a better quality of life (20,21). Although there are much evidence suggesting the efficacy of self-management in improving health outcomes in chronic diseases (16,17,19,21,22), little attention has been paid to the implementation of such programs specific to patients with sickle cell disease. The aim of this study was to determine the effectiveness of self-management programs on the quality of life for sickle cell patients, considering the high prevalence of the disease in the Khuzestan province and the need to control this chronic disease in order to improve quality of life for patients.

## Materials and methods

This research was a quasi-experimental (one-group before and after) study performed at the Thalassemia Clinic of Shafa Hospital affiliated to the Ahvaz Jundishapur University of Medical Sciences, Khuzestan, Iran. This study was approved by the Ethics Committee of Ahvaz University of Medical Sciences (Ethic Code ETH-739), and all patients gave their oral and written consent to participate in this study. Inclusion criteria for all people with sickle cell disease or sickle thalassemia aged over 18 years (due to the limited study population, the samples were the study population) were: ability to read and write, having a strong understanding of the Persian language, residing in the city of Ahvaz or having the opportunity to attend the sessions, and not suffering from a known mental illness. Participants were excluded from the study in case of lack of participation in individual and group training sessions (being absent for one session) and non-compliance with a practical program that was determined at monthly visits.

**Figure1:**
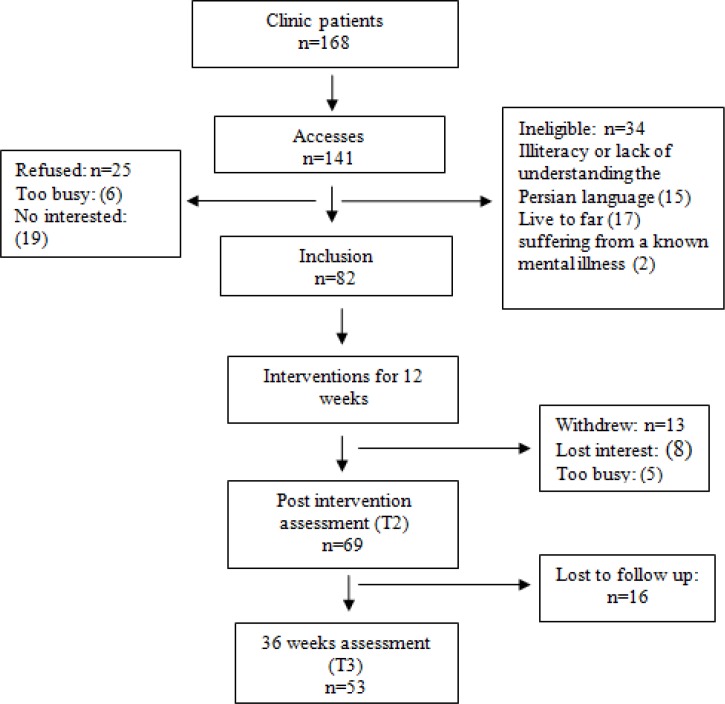
Progression of Participants

To access the samples, we extracted the medical records of all sickle cell patients aged over 18 years referring to the Thalassemia Clinic of Ahwaz Shafa Hospital during 2011 to 2013. Then, some descriptions of the purpose and outline of the study were provided by telephone to patients who were invited to participate in the study. Since despite numerous calls, the researcher did not have access to some of these patients. For better access to the entire study population, the researcher attended the clinic for six 6 months from February 2012 to June 2013, and the patients referring to the clinic were invited to participate in the research. Figure 1 shows a summary of how to access the samples.

The data gathering instruments included the demographic information questionnaire, the health behavior assessment form, and SF36 questionnaire. The first instrument consisted of eight questions (age, sex, marital status, level of education, type of sickle cell disease, ethnicity, occupation, and the number of hospitalizations due to pain crisis in the previous year). The behavioural health assessment form, including 13 questions, was set based on the guidelines available from reliable sources (23,24) and expert professors. This form was used for assessing needs and setting behavioural objectives.

The demographic information questionnaire and the behavioural health assessment form were researcher-made, for which the content validity method was used to determine the scientific validity of the instrument.

The Persian version of the SF-36 questionnaire was used in this study to assess the quality of life. It consisted of 36 questions that examine the eight QoL dimensions, including physical functioning, role-physical, bodily pain, general health, vitality, social functioning, role-emotional, and mental health. Each question has a continuous quantitative scale from 0 to 100, in which higher scores indicate better situation. This questionnaire is one of the most commonly used standard tools available at international level to assess health status and quality of life, and is the most common tool for measuring the quality of life in adult patients with sickle cell disease (25). It is a valid and reliable scale that has been used and standardized in various studies in Iran (26,27) and was completed in the intervention baseline (T1), the twelfth week (T2) and the thirty-sixth week (T3) by the participants.

In this study, a model called 5A self-management model (Assess, Advise, Agree, Assistant, and Arrange) was used to implement self-management program for patients with sickle cell disease. This model, presented by Whitlock and Glasgow, provides a useful framework for implementing evidence-based behaviour change interventions (23,24).

In the assess stage, patient's health behaviours, beliefs, and knowledge of the disease were evaluated by asking questions using the behavioral health assessment form. Furthermore, the results of experiments in patients' records were examined. These studies helped to assess needs and set behavioral objectives in the later stages of the model. At the advice stage, patients were informed of the abnormalities observed in the experiments and studies, as well as health risks. In addition, the benefits of behavioural change and its relationship with health, the consequences of the lack of control of the disease, and the benefits of disease control were clearly explained to the patients. At the agreement stage, appropriate behavioral objectives and behavior change methods were adjusted based on the interest and willingness of the patient, and were recorded in the record form of behavioral objectives and practical program. In this form a scale of one to ten was presented to the patients for each behavioral objective to determine their level of confidence in the implementation of the program, which had a significant role in raising the enthusiasm of patients. Then, the patients were asked to record their status on each of the objectives in the daily record checklists for 12 weeks. At this stage, in addition to behavioral objectives set for each patient, an agreement was made with all patients for two practical programs, including daily fluid intake and daily walks. In order to formulate behavioural objectives, the participants were asked to record, using diaries, the cases that caused attacks of pain and then deliver the diaries in the following month. In addition, another agreement was made between the researcher and each patient in relation to participation in the individual and group counselling sessions and plural for monthly visits. The three aforementioned stages were performed for each patient in a session of about two hours in a completely individual manner. Implementing the first three stages, the fourth and fifth stages simultaneously began.

In the assistance stage, an individual training session was organized for each patient in the second week of the intervention while he/she was taught in a completely individual manner on pain crisis, warning signs, the cases that need referral to the emergency department, how to relieve the pain using home treatment (massage, the use of liquids, hot shower, the use of hot water bag, and rest), as well as cognitive and behavioral techniques to deal with the pain (including relaxation, deep breathing, distraction techniques such as mental imagery, and repeating positive phrases to adapt to pain). Patients were asked to perform deep breathing and relaxation exercises twice a day, each time for 10 to 15 minutes, and check them in the daily record checklists. A group session of 10-15 people was held in the third week when the patients were informed about the disease and its management through slides and images. The session lasted about four hours, at the end of which a brochure and a CD containing the training materials presented at the session were provided to each patient. Based on patients' needs, individual training sessions were organized again between the fourth and twelfth weeks of intervention. In addition, two small group training sessions were held in the form of homogeneous groups of 3-4 persons, during which the required provision of training was taught to patients. Moreover, they were asked to discuss their successful experiences on the pain control or other problems that they could more easily adopt the successful experiences of each other. At this stage, patients were taught on problem solving techniques, and were helped to identify barriers to behavior change, and were acquainted with to overcome those barriers. Adequate feedback was provided and enough time was allocated answering patients questions. The assistance stage continued throughout the follow-up period.

In the first intervention week after the agreement stage, a 36 week follow-up phase began that was conducted in two phases over a period of 12 weeks (intervention period) and 24 weeks (follow-up after intervention). During the first 12 weeks, the patient's progress was checked by telephone once a week. To establish continuous interaction, the telephone number of the researcher was provided to the patients. Patients were also visited every 4 weeks. During the monthly visit sessions that lasted about half an hour, the practical programs and related checklists were examined and necessary changes in objectives and practical programs, if needed, were made upon a further agreement. In the 24 weeks follow-up after intervention, a monthly communication with patients was made by telephone to evaluate the feedback of training, and provide necessary instruction. Whenever an unplanned visit was needed, the researcher was present at the clinic.

**Statistical Analysis:**

The obtained data were entered into the statistical software SPSS v.19. We used descriptive statistics to determine the frequency, mean and standard deviation, and paired t-test and Wilcoxon test to compare the mean score of eight dimensions and the total QoL score before and after the intervention. The Hotelling's T-squared multivariate test was also applied to compare the structure of life quality before and after the intervention. The repeated measures test was used to compare the eight dimensions and the total QoL score at the baseline, twelfth and thirty-sixth week of the intervention.

## Results

Based on the study findings, the average age of participants was 25.84±7.23. There were 52 women (75.4%) and 17 men (24.6%). Other demographic information is shown in Table 1.

The comparison between the QoL scores in patients with sickle cell disease before and after the intervention revealed that the mean score of eight QoL dimensions as well as the total QoL score after intervention had increased compared to those before the intervention, and paired t-test and Wilcoxon test indicated a significant difference (P<0.001) (Table 2). The effect of intervention on the structure of life quality was assessed through Hotelling's T-squared test and showed significant differences (P<0.001) (Table 3). ANOVA with repeated measures, which was performed on 53 patients, showed that the mean score of eight dimensions and the total QoL score were also significantly improved in the twelfth week and thirty-sixth week, compared to the intervention baseline (P<0.05), but compared to the twelfth week, it decreased in the thirty-sixth week. Although there was no significant decrease in more dimensions, elaborate this decrease was significant in the dimensions of role-physical, general health, and total QoL score (Table 4).

**Table I:** Descriptive data for demographic variables

	**Participants(n=69)** **n (%)**
Gender	
Female Male	**52(75.4)** **17(24.6)**
Marital Status	
Married single Divorced	**20(29)** **47(68.1)** **2(2.9)**
Education	
Less than high school High school diploma Some college Bachelor’s degree	**35(50.7)** **24 (34.8)** **3(4.3)** **7(10.1)**
Employed full or part time Un employed	**16(23.1)** **53(76.9)**
Sickle cell disease genotype	
HbSS Sickle Beta Thalassemia	**44(63.8)** **25(36.2)**
Ethnicity	
Arab Farse	**67(97.1)** **2(2.9)**

.

**TableII:** Comparison between changes in Quality of life before and after the intervention in patients with sickle cell disease

**Total Sample (** ***N *** **= 69)**			
**SF-36 Domains**	Baseline	12 Weeks	P
	M±SD	M±SD	
**Physical functioning** 1	65.14±22.07	88/04±12.52	<.001
**Role limitations-physical** 1	31.56±36.25	76.45±31.47	<.001
**Role limitations-emotional** 1	36.23±41.12	71.50±35.82	<.001
**Social functioning** 1	56.70±24.68	85.33±15.74	<.001
**Pain** 1	52.75±28/.5	76.78±21/03	<.001
**Energy/ fatigue** 2	47.17±19.35	71.75±16.21	<.001
**Emotional well being** 2	54.90±20.63	72.99±16.62	<.001
**General health** 2	42.97±20.69	76.52±16.16	<.001
**Total** 1	50.94±15.69	78.84±11.82	<.001

1 :Paired t test

2 : Wilcoxon test

**Table III:** Simultaneous Comparison of quality of life before and after intervention

**Quality of life**	**Hoteling T2**		
	value	F	**P**
	28.608	461.309	**<.001**

**Table IV:** Comparison of Quality of life means scores at different stages in patients with sickle cell disease

**Total Sample (** ***N *** **= 53)**				**Comparisons Across Time**		
**SF-36 Domains**	Baseline T1	12 Weeks T2	36 Weeks T3	T1 to T2	T1 to T3	T2 to T3
	M±SD	M±SD	M±SD	P	P	P
**Physical functioning**	68.4±20.51	89.72±9.67	87.36±15.33	<.001	<.001	.56
**Role limitations-physical**	34.96±37.40	79.72±28.61	61.32±37.84	<.001	<.001	.004
**Role limitations-emotional **	36.48±40.96	72.33±35.64	71.07±34.61	<.001	<.001	.9
**Social functioning **	56.13±25.19	88.44±13.83	83.25±19.9	<.001	<.001	.2
**Pain **	52.45±28.39	78.11±19.93	68.02±28.73	<.001	.02	.1
**Energy/ fatigue**	48.02±18.51	74.35±15.70	71.13±18.38	<.001	<.001	.64
**Emotional well being**	55.70±20.72	75.25±16.43	74.19±18.73	<.001	<.001	.9
**General health**	42.92±20.76	77.26±15.76	70.19±20.23	<.001	<.001	.01
**Total**	52.44±15.20	80.77±10.73	75.37±15.69	<.001	<.001	.02

## Discussion

This study aims to investigate the effect of self-management on life quality of sickle cell patients. Since during their lifetime, sickle cell patients suffer from complications such as physical, mental, and social disorders which clearly impair their quality of life, the implementation of self-management programs to improve health outcomes has always been emphasized by researchers (16,28). Based on Previous studies sickle cell patients are faced with limitations in different dimensions of quality of life, including physical and mental health (15,29-31). The results of current studies showed that the mean QOL scores were significantly lower in all dimensions, especially role-physical, mental problems, vitality as well as general health, and was significantly improved after implementing self-management program. Some of the characteristics of the intervention also appeared to be effective in promoting the quality of life of patients with sickle cell disease: a detailed evaluation of patients, and the design of self-management program based on each patient's needs, personal face to face training, agreement with the patient on behavioral objectives and completion of checklists for practical program, group discussions to share experiences, establishing ongoing communication with the patients creating necessary changes in practical programs in order to achieve the behavioral objectives, individual consultations, and recommendations for in-home pain management with the training of cognitive and behavioral techniques to deal with the pain. Although there are a number of studies on the implementation of self-management programs for sickle cell patients, their results are not comparable with the results of the present study. However, studies conducted by Edward in Colombia and Anie in London (32,33) are similar to this study in terms of behavioral interventions. The first study, which investigate the efficacy of an intervention for pain management based on cognitive behavioral management strategies, showed statistically significant differences between behavioral intervention group and the patient education group in terms of QOL mean scores in eight dimensions. However, the mean scores of eight QoL dimensions after intervention were reduced in both groups. Edward considered various factors that cause this reduction, such as small sample size, the selection of participants among patients admitted to an acute care facility which led to the selection of patient with severe disease, insufficient interactions between nurses and patients, and the lack of practical programs in their intervention; (32) in contrast to aforementioned study, current study favoured extensive interaction between researchers and patients in the intervention period, continual follow-ups and monthly visits as well as establishment of numerous individual and group sessions to suit the needs of patients. In line with previous studies (34), the findings of this study emphasize the role of health care provider in an environment where patients can be actively involved in their own self-management as an element of success. The development of practical programs based on the patients' needs is one of the main features of the 5A model, which played a very important role in patients' adherence to self-care. The study of Anie et al, which was of quasi-experimental (one-group before and after) design, showed an increase in QOL mean scores in 8 dimensions and the total score after the intervention implementation which is consistent with the results of the present study. However, significant differences in the dimensions of vitality and general health were merely reported (33). The insignificant nature of other QOL dimensions in the above study may be attributed to its small sample size compared to our study. The provision of training was the same for all patients in both studies, while there are many factors that distinguish the 5A's behavior change model from the behavior change model used in the study of Anie et al including need assessment and goal setting based on the needs of patients, guidance on the behaviors to be changed, and agreement with patients to perform health behaviours (during which the patient is actively involved in the development of his/her self-management program). Previous studies have shown that patient-cantered care, supporting sickle cell patient's independence, as well as participatory decision-making are associated with better self-management in sickle cell patients (34). The results of our study are consistent by the findings of another study in patients with diabetes. Sickle cell disease and diabetes are both chronic diseases associated with many serious comorbidities and could benefit from self-management programs. The results of Kanna's study et al. on diabetics, showed that the implementation of a self-management program had been effective in increasing QOL in patients with diabetes (35). In comparison with 12th week, life quality of patients decreased during the thirty-sixth week, nevertheless, compared to base line, life quality even in this week had significant promotion. This decrease can be attributed to reduced follow-ups and limited contact with patients at 24 weeks after the implementation of the intervention.

**Implication of the study:**

Given that the disease is chronic with an erosive effect that over time can affect quality of life, continuous follow-up is required for participants who may need additional and continuing support to be able successfully manage their situation and achieve long term promotion in self-management behaviors. Other studies have also highlighted that increase in contact time with patients during self-management follow-up programs can led to greater beneficial effects (36).

**Limitation of the study:**

Since there was no control group, the obtained findings are not definitive and further studies with a well-designed randomized control trial and evaluation of the long term effects of such programs are recommended.

**Conclusion:**

Although self-management programs for sickle cell patients have received little attention by researchers, our results show that if a self-management program is designed based on the needs of sickle cell patients, it can be effective in motivating them to change behavior and thus promote the quality of life. In general, the findings of the study revealed that the 5A self-management model improve quality of life in sickle cell patients. We would therefore recommend the use of 5A model as an easy option for nurses to help the patients.
